# Fabrication and characterization of up-converting β-NaYF_4_:Er^3+^,Yb^3+^@NaYF_4_ core–shell nanoparticles for temperature sensing applications

**DOI:** 10.1038/s41598-020-71606-6

**Published:** 2020-09-04

**Authors:** Lam Thi Kieu Giang, Karolina Trejgis, Lukasz Marciniak, Nguyen Vu, Le Quoc Minh

**Affiliations:** 1 Institute of Materials Science, Vietnam Academy of Science and Technology, 18 Hoang Quoc Viet Road, Cau Giay District, Hanoi, Vietnam; 2 Graduate University of Science and Technology, Vietnam Academy of Science and Technology, 18 Hoang Quoc Viet Road, Cau Giay District, Hanoi, Vietnam; 3 Institute of Low Temperature and Structural Research, Polish Academy of Sciences, ul. Okólna 2, 50-422 Wrocław, Poland; 4 Duy Tan University, 7/25 Quang Trung, Da Nang, Vietnam

**Keywords:** Materials science, Nanoscience and technology

## Abstract

This paper presents the use of soft template method to synthesize core and core–shell up-converting nanoparticles usefull for temperature sensing applications. Based on the stock solutions of core β-NaYF_4_:Er^3+^,Yb^3+^ nanoparticles and involving soft template method without any additional process of surface functionalization, it is possible to directly design the core–shell β-NaYF_4_:Er^3+^,Yb^3+^@NaYF_4_ nanoparticles, which can be perfectly dispersed in cyclohexane and surfactants like oleic acid (OA), triethanolamine (TEA) or Cetyltrimethylammonium bromide (CTAB). The morphological, crystalline and elemental characteristics of samples were investigated by Field Emission Scanning Electron Microscopy, X-Ray Diffraction, High Resolution Transmission Electron Microscopy, Selected Area Electron Diffraction patterns and Energy-Dispersive X-Ray Spectroscopy (EDX) measurements. The results showed that the synthesized NaYF_4_:Er^3+^,Yb^3+^@NaYF_4_ core–shell nanoparticles have roughly spherical shape, pure hexagonal β phase with core size of about 35 ± 5 nm and shell thickness of about 40 ± 5 nm. It has been shown that the coating of the β-NaYF_4_:Er^3+^,Yb^3+^ core with NaYF_4_ shell layer enables to enhance the green upconversion (UC) emission intensities in respect to red one. Under 976 nm excitation, the synthesized β-NaYF_4_:2%Er^3+^,19%Yb^3+^@NaYF_4_ core–shell nanoparticles revealed three strong emission bands at 520 nm, 545 nm and 650 nm corresponding to ^2^H_11/2_, ^4^S_3/2_ and ^4^F_9/2_ to ^4^I_15/2_ transitions of Er^3+^ ions with the lifetimes of 215, 193 and 474 µs, respectively. The calculated CIE chromaticity coordinates proved that the emission colour of core–shell nanoparticles was changed from red into yellowish green upon increasing the power density of the 976 nm laser from 0.73 to 9.95 W/cm^2^. The calculated slopes indicated that in the β-NaYF_4_:2%Er^3+^,19%Yb^3+^@NaYF_4_ core–shell nanoparticles, two-photon and three-photon UC processes took place simultaneously. Although the former one is similar as in the case of β-NaYF_4_:Er^3+^,Yb^3+^ bare core nanoparticles, the latter one, three-photon UC process for green emission occurs, due to cross relaxation processes of two Er^3+^ ions only within nanoparticles with core–shell architecture. Moreover, the energy difference between the ^2^H_11/2_ and ^4^S_3/2_ levels and associated constant of NaYF_4_@NaYF_4_ host lattice were determined and they reached ~ 813 cm^−1^ and 14.27 (r^2^ = 0.998), respectively. In order to investigate the suitability of nanoparticles for optical temperature sensing, the emission spectra were measured in a wide temperature range from 158 to 298 K. An exceptionally high value of relative sensitivity was obtained at 158 K and it amounted to 4.25% K^−1^. Further temperature increase resulted in gradual decrease of relative sensitivity, however, it maintained a high value > 1% K^−1^ in the entire analyzed temperature range.

## Introduction

Temperature is an important physical parameter which governs many fields of science, such as medicine, biology, microelectronics, physics, and chemistry^1^^.^ Therefore, many methods of temperature measurements, both contact and non-contact, have been developed based on the mechanical, electrical or optical characteristics^2–4^. However, to determine the temperature of small, moving or inaccessible objects, the non-contact luminescent thermometry which exploits thermal changes of spectral or temporal properties of the phosphor attached to the object is a preferred technique^4,5^. One of the most extensively investigated group of lanthanide-based luminescent thermometers are those which for temperature sensing, take advantage of thermally coupled energy levels, like in the case of Er^3+^, Tm^3+^, Ho^3+^, Pr^3+^ or Eu^3+^^6–10^. In recent years, the upconversion luminescence of rare-earth ions (RE^3+^) doped fluoride nanomaterials has received much attention due to their potential applications in various fields such as bioimaging, biolabeling, photodynamic therapy, as drug carriers, optical temperature sensors, etc.^11–18^. In the case of the optical temperature sensing applications, researchers focused especially on the sodium yttrium fluoride nanoparticles doped or co-doped with rare earth ions (NaYF_4_:RE^3+^ nanoparticles)^19–24^, among which the NaYF_4_:Er^3+^, Yb^3+^ nanoparticles were regarded as one of the most efficient upconversion materials for temperature sensing due to their unique luminescence properties and the large energy gap between two thermally coupled ^2^H_11/2_ and ^4^S_3/2_ energy levels^25–27^. One of the most important parameter of temperature sensor is its relative sensitivity to temperature changes which verify its quality as well as range of their applications. The improvement of the thermometer sensitivity can be influenced by many factors, e.g. the effect of the phase of β-NaYF_4_:Er^3+^,Yb^3+^ and α-NaYF_4_:Er^3+^,Yb^3+^ nanoparticles on the relative sensitivity has been compared. Under the same experimental conditions, i.e. under 980 nm excitation and in a wide temperature range from 328 to 550 K, the β-NaYF_4_:Er^3+^,Yb^3+^ nanoparticles revealed significantly higher sensitivities of 0.42% K^−1^ and 0.466% K^−1^ at 328 K and 550 K^26^, respectively than α-phase counterpart^27^. This confirms the importance of conscious synthesis process for the intentional obtaining of highly temperature sensitive nanomaterials and explains the reason for using beta phase nanomaterials in our research.

In our previous research the upconversion (UC) emission properties of uncoated β-NaYF_4_:Er^3+^,Yb^3+^ nanoparticles under 976 nm excitation have been investigated. In that case, emission spectra consisted mainly of red emission band at 650 nm, while the green part of spectrum covering the bands at 520 nm and 545 nm was very weak^28^. In order to enhance the green emission intensities used for temperature sensing applications, in this study a β-NaYF_4_:Er^3+^,Yb^3+^ nanoparticles coated with NaYF_4_ shell layer are presented. These core–shell nanoparticles were synthesized through the slightly modified soft template method reported previously^28^. The detailed comparison of UC emission properties under 976 nm excitation with power density changed from 0.73 to 9.95 W/cm^2^, the lifetimes and CIE chromaticity coordinates as well as emission mechanisms of uncoated β-NaYF_4_:Er^3+^,Yb^3+^ nanoparticles and NaYF_4_:Er^3+^, Yb^3+^@NaYF_4_ core–shell nanoparticles was also made. Moreover, the luminescence intensity ratio (LIR) of thermally coupled ^2^H_11/2_ and ^4^S_3/2_ excited states as a function of temperature ranging from 158 to 320 K was investigated. Based on the values of LIR, both the absolute and relative thermal sensitivities were determined. The maximal value of relative sensitivity reached 4.25% K^−1^ (at 158 K), confirming great suitability of these NaYF_4_:Er^3+^, Yb^3+^@NaYF_4_ core–shell nanoparticles for optical thermometry.

## Experimental

### Materials

The rare earth acetate hydrate salt precursors (RE(CH_3_COO)_3_.xH_2_O, 99.9%) including yttrium (III) acetate hydrate (Y(CH_3_COO)_3_.xH_2_O, 99.9%), ytterbium(III) acetate hydrate (Yb(CH_3_COO)_3_.xH_2_O, 99.9%), erbium(III) acetate hydrate (Er(CH_3_COO)_3_.xH_2_O, 99.9%) were purchased from Sigma-Aldrich. The oleic acid (OA, CH_3_(CH_2_)_7_CH = CH(CH_2_)_7_COOH, 99%), ammonium fluoride (NH_4_F, 98%), sodium acetate (CH_3_COONa, 99%), isopropanol (IPA, (CH_3_)_2_CHOH, 99%), cyclohexane (C_6_H_12_, 99%) and methanol (CH_3_OH, 99%) were purchased from Merck.

### Synthesis methods

**Figure 1:**
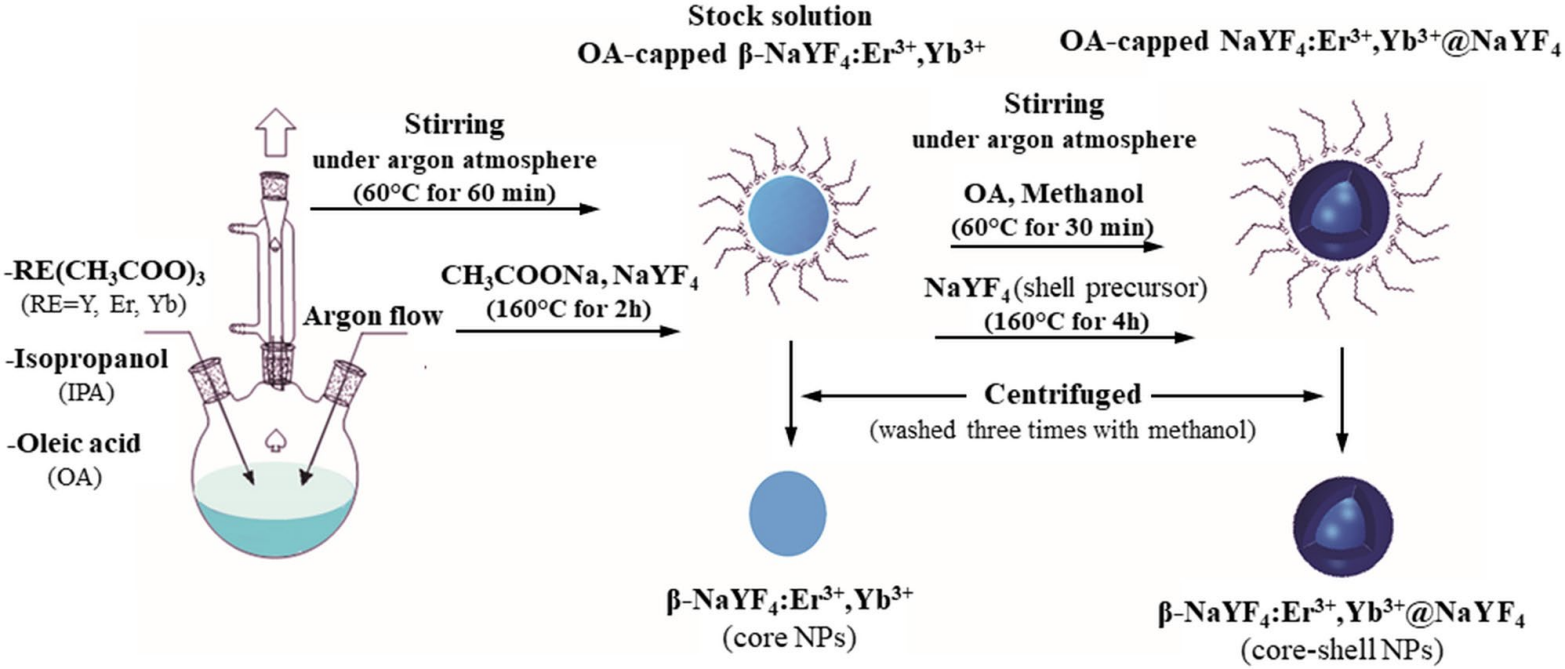
Schematic diagram of preparation of β-NaYF_4_:Er^3+^,Yb^3+^ CNP and β-NaYF_4_:Er^3+^,Yb^3+^@NaYF_4_ CSNP using soft-template method.

The β-NaYF_4_ co-doped with 2% mol of Er^3+^ and 19% mol of Yb^3+^ core nanoparticles (β-NaYF_4_:Er^3+^,Yb^3+^ CNP) and β-NaYF_4_@NaYF_4_ co-doped 2% mol of Er^3+^ and 19% mol of Yb^3+^ core–shell nanoparticles (β-NaYF_4_:Er^3+^,Yb^3+^@NaYF_4_ CSNP) were prepared by soft-template method as shown in Fig. 1.

#### Synthesis of β-NaYF_4_:Er^3+^,Yb^3+^ CNP

The β-NaYF_4_:Er^3+^,Yb^3+^ CNP were prepared via the slightly modified soft template method reported previously^28^. Yttrium acetate hydrate (0.79 mmol), erbium acetate hydrate (0.02 mmol) and ytterbium acetate tetrahydrate (0.19 mmol) were dispersed in 50 ml solution containing 10 ml IPA and 15 ml OA by ultrasonic cleaner for 30 min. To form a homogeneous solution, the mixture were stirred at 60 °C for 60 min under argon atmosphere. Then the mixed solution containing 1.1 mmol sodium acetate, 4.0 mmol ammonium fluoride and 10 ml of methanol was slowly added to the solution under magnetic stirring for another 30 min. Finally, the reaction mixture was heated up to 160 °C and stirred for 2 h under argon atmosphere, then cooled down to room temperature in the ice water bath to obtain the stock solution of β-NaYF_4_:Er^3+^,Yb^3+^ CNP.

#### Preparation of the shell precursor solution of NaYF_4_

The shell precursor solution of NaYF_4_ was prepared analogously to the β-NaYF_4_:Er^3+^,Yb^3+^ CNP as described above however using 1.0 mmol yttrium acetate hydrate, without addition of erbium and ytterbium acetates.

#### Synthesis of β-NaYF_4_:Er^3+^,Yb^3+^@NaYF_4_ CSNP

Dispersion of 30 ml stock solution of prepared β-NaYF_4_:Er^3+^,Yb^3+^ CNP (described above) in mixed solution of 10 ml oleic acid and 15 ml methanol was carried out using ultrasonic bath for 30 min. Then, the homogeneous mixture of β-NaYF_4_:Er^3+^,Yb^3+^ CNP with oleate ligands capping the surface was achieved via stirring at 60 °C for 30 min under argon atmosphere. Finally, during another 30 min lasting stirring at 60 °C under argon atmosphere the 30 ml of the shell precursor solution of NaYF_4_ was slowly added to the mixture and then heated up to 160 °C for 4 h, then cooled down to room temperature in the ice water bath to obtain the solution of β-NaYF_4_:Er^3+^,Yb^3+^@NaYF_4_ CSNP.

The precipitated β-NaYF_4_:Er^3+^,Yb^3+^ CNP and β-NaYF_4_:Er^3+^,Yb^3+^@NaYF_4_ CSNP were centrifuged and washed three times with methanol, then dried at 60 °C and dispersed in cyclohexane.

### Characterization

The morphology of synthesized β-NaYF_4_:Er^3+^,Yb^3+^ CNP and β-NaYF_4_:Er^3+^,Yb^3+^@NaYF_4_ CSNP were carried out by Field Emission Scanning Electron Microscope (FESEM, Hitachi S-4800). Transmission Electron Microscopy images were recorded using Tecnai 20 D2094 X-Twin Microscope operating at 200 kV, in which, the samples were prepared by dispersion in ethanol with an ultrasonic bath for 20 min, then put on the copper grids. Selected Area Electron Diffraction (SAED) patterns and Energy Dispersive X-ray Spectroscopy (EDX) elementals mapping were carried out using Tecnai 20 D2094 X-Twin Microscope operating at 200 kV. Structural analysis of samples was determined by X-Ray Diffraction (PANalytical X’Pert Pro Powder Diffractometer) using Cu K_α_ radiation (λ = 1.54060 Å) in the 2θ range from 10° to 80°.

The Fourier Transform Infrared Spectroscopy (FTIR) analysis was recorded using a Thermo Nicolet NEXUS 670 FTIR (USA) in a KBr suspension in the region of 4000 to 400 cm^−1^.

The upconversion emission spectra were measured with a Jobin–Yvon HR1000 monochromator, equipped with a charge-coupled device (CCD) camera at temperature in range from 158 to 320 K by the use of THMS600 heating–cooling stage from Linkam and using a 976 nm laser diode of power density changed in a range from 0.73 to 9.95 W/cm^2^. The upconversion luminescence decay curves were measured with a LeCroy WaveSurfer 400 oscilloscope.

## Results and discussion

**Figure 2:**
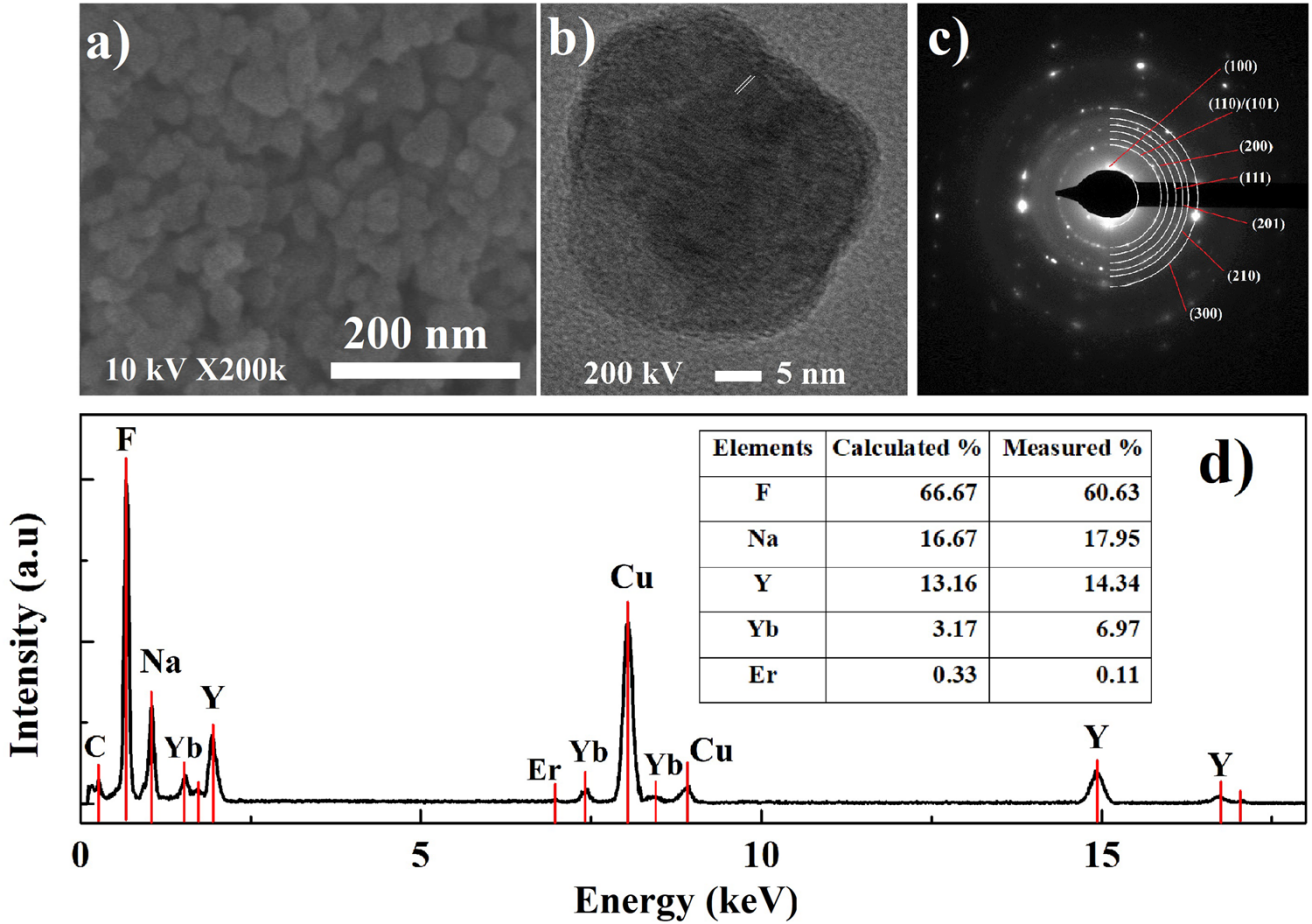
Characterization of synthesized NaYF_4_:Er^3+^,Yb^3+^ CNP: (**a**) FESEM and (**b**) HR-TEM images, (**c**) SAED pattern and (**d**) EDX spectrum corresponding to the isometric nanoparticles in (**c**).

**Figure 3:**
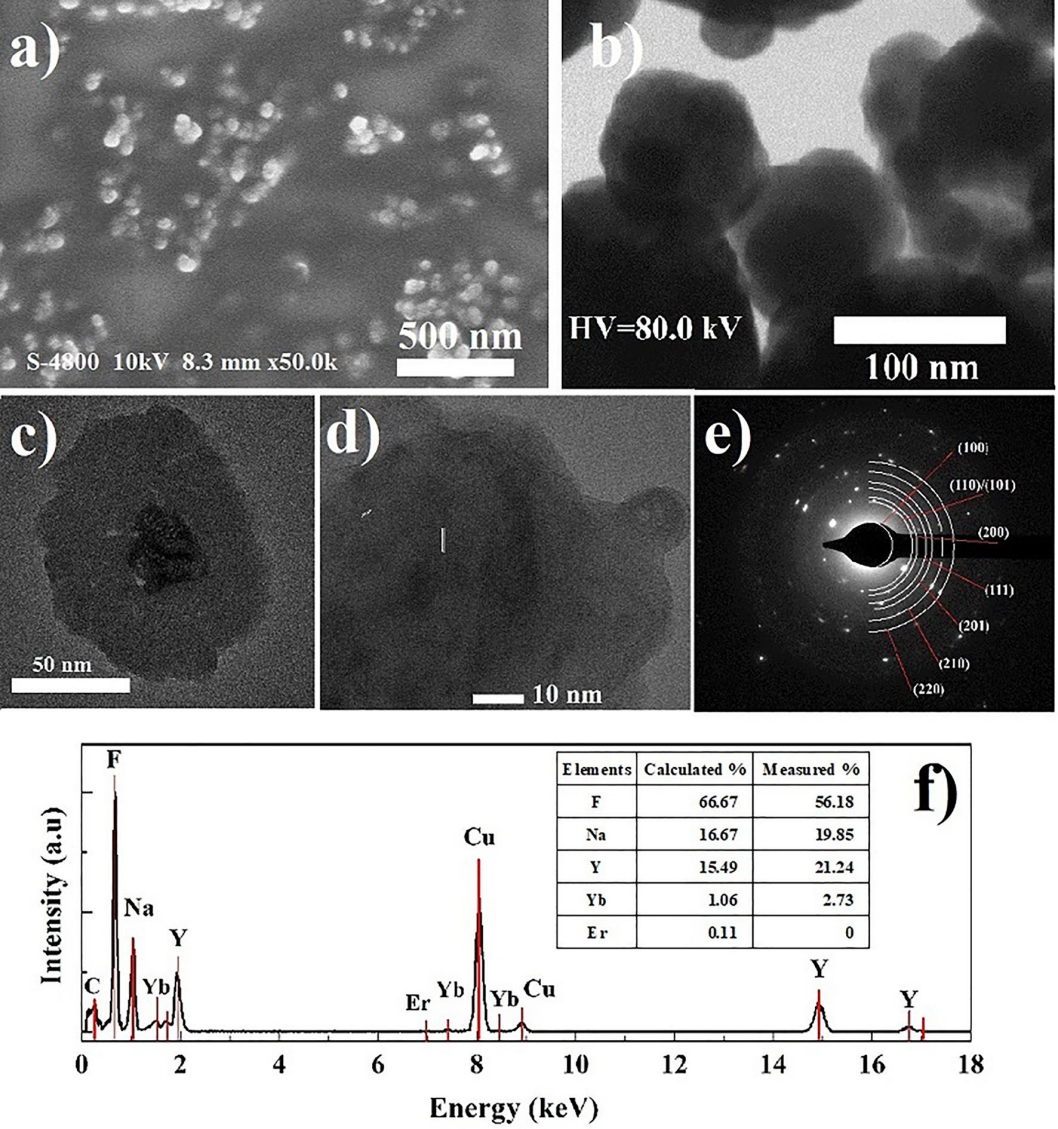
Characterization of NaYF_4_:Er^3+^,Yb^3+^@NaYF_4_ CSNP: (**a**) FE-SEM, (**b**) TEM, (**c**, **d**) HR-TEM images, (**e**) SAED pattern and (**f**) EDX spectrum corresponding to the isometric nanoparticles (**e**).

**Figure 4:**
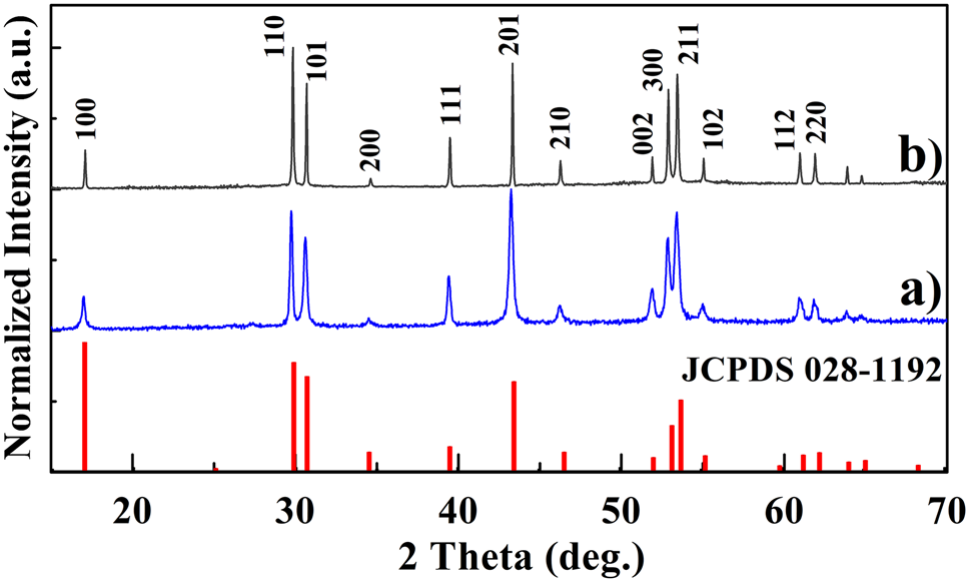
The normalized XRD patterns of synthesized NaYF_4_:Er^3+^,Yb^3+^CNP (**a**) and NaYF_4_:Er^3+^,Yb^3+^@NaYF_4_ CSNP (**b**) compared to standard data of hexagonal β-NaYF_4_ phase (JCPDS card No. 028-1192).

Comparison of morphological and structural properties of the uncoated nanoparticles (NaYF_4_:Er^3+^,Yb^3+^ CNP) and core–shell structures (NaYF_4_:Er^3+^,Yb^3+^@NaYF_4_ CSNP) is shown in Figs. 2, 3 and 4. As can be seen in the FESEM image (Fig. 2a), the synthesized NaYF_4_:Er^3+^,Yb^3+^ CNP were roughly spherical in shape with sizes of around 35 ± 5 nm. The HR-TEM image in Fig. 2b presents a representative nanoparticle with size of 36 nm and lattice distance of 0.3 nm corresponding to d-spacing for the (110) lattice plane of hexagonal β phase of NaYF_4_ structures, suggesting that the [110] is the preferred growth direction of the NaYF_4_:Er^3+^,Yb^3+^ CNP. The clear difference between core and shell part of the nanoparticle observed in TEM images may results from the difference in the porosity of the particular parts of the nanocrystal. In addition, the obtained SEAD pattern in Fig. 2c indicates the diffraction rings which correspond to the (100), (110), (101), (200), (111), (201), (210) and (300) planes of the synthesized β-NaYF_4_ nanoparticles. The EDX spectrum analysis shows the major peaks at energy around 0.68, 1.04, 1.91 and 8.04 keV corresponding to F, Na, Y of synthesized NaYF_4_:Er^3+^,Yb^3+^ CNP and Cu elements of copper grid (Fig. 2d). The minor intensity peaks observed at energy around 1.54, 7.39, 8.42, 14.9 and 16.73 keV have proven the presence of Yb^3+^ and Y^3+^ ions in the samples. The presence of Er^3+^ ions at energy around 6.9 keV is almost invisible which is due to the low concentration of Er^3+^ ions in the material.

The characteristic properties of NaYF_4_:Er^3+^,Yb^3+^@NaYF_4_ CSNP are presented in Fig. 3. As can be observed in Fig. 3a–c, after coating the surface of NaYF_4_:Er^3+^,Yb^3+^ CNP with a shell layer of NaYF_4_, the synthesized NaYF_4_:Er^3+^,Yb^3+^@NaYF_4_ samples revealed also roughly spherical shape however, with obviously larger average size than that of the above-mentioned NaYF_4_:Er^3+^,Yb^3+^ CNP precursors. The diameter of the core–shell nanoparticles was on average 100 ± 10 nm. It can be clearly seen that the NaYF_4_ shell layer with thickness about 40 nm covered the entire surface of NaYF_4_:Er^3+^,Yb^3+^ CNP, indicating the formation of core–shell structure in the synthesized NaYF_4_:Er^3+^,Yb^3+^@NaYF_4_ CSNP. In particular, the HR-TEM image (Fig. 3d) has shown that the lattice distances were equal to 0.3 nm as in the case of uncovered NPs and 0.5 nm corresponding to d-spacing for the (110) and (100) lattice planes of β-NaYF_4_ nanoparticles. It suggests that the [110] growth direction is preferred both in the case of uncovered NaYF_4_:Er^3+^,Yb^3+^ CNP as well as in the case of core–shell NaYF_4_:Er^3+^,Yb^3+^@NaYF_4_ CSNP. Figure 3e displays the diffraction rings corresponding to the (100), (110), (101), (200), (111), (201), (210) and (220) planes of the β-NaYF_4_ nanoparticles thus confirming the formation of hexagonal β phase of synthesized NaYF_4_:Er^3+^,Yb^3+^@NaYF_4_ CSNP. Furthermore, the results of EDX spectrum analysis confirmed the presence of F, Na, Y host elements which were related to the major peaks at energy around 0.67, 1.04, 1.92 and 8.04 keV (Fig. 3f). However, the intensities of peaks corresponding to Yb element at energy around 1.54, 7.39 and 8.42 keV in the NaYF_4_:Er^3+^,Yb^3+^@NaYF_4_ CSNP were much lower compared with the NaYF_4_:Er^3+^,Yb^3+^ CNP, suggesting as expected a decrease of Yb^3+^/NaYF_4_ ratio in the NaYF_4_:Er^3+^,Yb^3+^@NaYF_4_ CSNP since the ratio of Yb^3+^ ions to the total amount of ions is lower in the case of CSNP in respect to the CNP counterpart due to the additional shell layer with no Yb^3+^ ions. The presence of Er^3+^ ions was not observed in the NaYF_4_:Er^3+^,Yb^3+^@NaYF_4_ CSNP due to the Er^3+^ concentration below the detection limit of the measurement.

The normalized XRD patterns of NaYF_4_:Er^3+^,Yb^3+^ CNP and NaYF_4_:Er^3+^,Yb^3+^@NaYF_4_ CSNP are shown in Fig. 4. The diffraction peaks of samples perfectly matched with the standard reference pattern of hexagonal β phase of NaYF_4_ (JCPDS card, No. 028-1192) which confirmed the high purity and crystallization of synthesized NaYF_4_:Er^3+^,Yb^3+^ CNP and NaYF_4_:Er^3+^,Yb^3+^@NaYF_4_ CSNP.

The average crystallite size D of samples was calculated using Scherrer formula^29^:

1 $${\text{D}} = \frac{k\lambda}{\beta\cos\theta }$$

where λ is the X-ray wavelength, β is the full width at half maximum, θ is the diffraction angle and k is a constant. The determined D values were equal 36 nm and 85 nm for NaYF_4_:Er^3+^,Yb^3+^ CNP (pattern a) and β-NaYF_4_:Er^3+^,Yb^3+^@NaYF_4_ CSNP (pattern b), respectively^29^. The results were entirely consistent with the SEM and TEM images presented above in Figs. 2 and 3.

**Figure 5:**
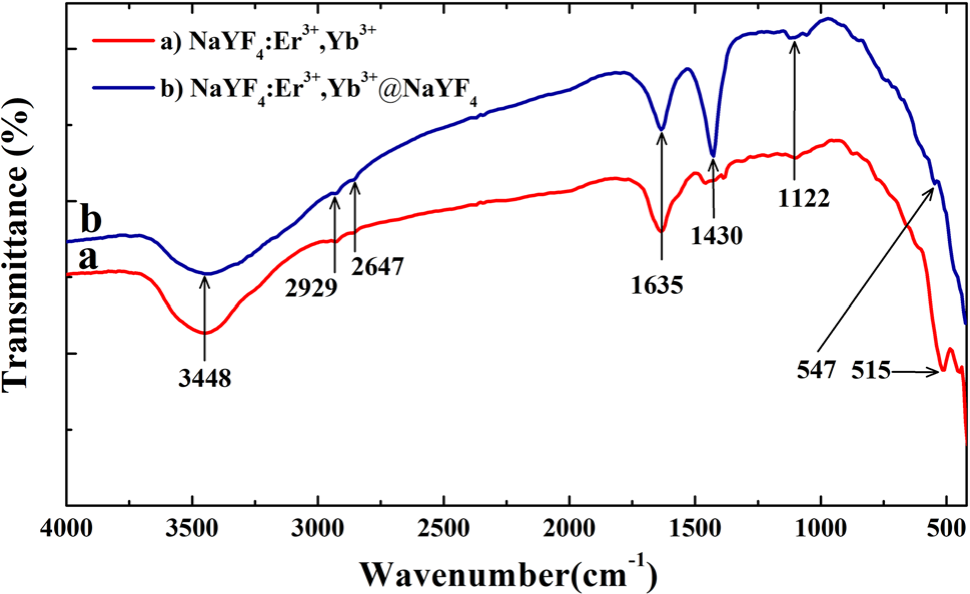
The FTIR spectra of: (**a**) β-NaYF_4_:Er^3+^,Yb^3+^ CNP and (**b**) β-NaYF_4_:Er^3+^,Yb^3+^@NaYF_4_ CSNP in the wavenumber range of 4000 to 400 cm^−1^.

The ligand binding on the surface of β-NaYF_4_:Er^3+^,Yb^3+^ CNP and β-NaYF_4_:Er^3+^,Yb^3+^@NaYF_4_ CSNP were investigated by FTIR spectrum in the wavenumber range from 4000 to 400 cm^−1^ (Fig. 5). As can be seen, in both core and core–shell samples, a broad absorption peak of the O–H stretching vibrations due to intermolecular hydrogen bonding at around 3448 cm^−1^ was observed^30^. The weak absorption peaks at 2929 cm^−1^, 2647 cm^−1^ corresponding to the asymmetric and symmetric stretching vibrations of the long alkyl chains (= CH(CH_2_)_n_ groups) of oleic acid can be observed confirming its residual presence on the surface of β-NaYF_4_:Er^3+^,Yb^3+^ CNP and β-NaYF_4_:Er^3+^,Yb^3+^@NaYF_4_ CSNP. The broad bands at about 1635 cm^−1^ and 1122 cm^−1^ have been assigned to the presence of asymmetric and symmetric C = O stretching vibration of carboxylic acid salts on the surface of β-NaYF_4_:Er^3+^,Yb^3+^ CNP and β-NaYF_4_:Er^3+^,Yb^3+^@NaYF_4_ CSNP^31^ and the peaks at 547 cm^−1^ and 515 cm^−1^ might be a result of a presence of the RE^3+^–F stretching vibrations. Exceptionally, a strong band at 1430 cm^−1^ related to O–H bending vibration of carboxyl group was observed only in the β-NaYF_4_:Er^3+^,Yb^3+^@NaYF_4_ CSNP^17,30^. Therefore, the presence of carboxylic acid groups in the synthesized β-NaYF_4_:Er^3+^,Yb^3+^@NaYF_4_ CSNP is suspected^30^.

In order to study the impact of Er^3+^ and Yb^3+^ ions as well as the energy transfer mechanisms in synthesized β-NaYF_4_:Er^3+^,Yb^3+^ CNP and β-NaYF_4_:Er^3+^,Yb^3+^@NaYF_4_ CSNP on their spectroscopic properties, the dependence of UC luminescence intensity on the 976 nm excitation (power density changed in the range from 0.73 to 9.95 W/cm^2^) have been measured at room temperature. As it is well known, in the low excitation density regime, the UC emission intensity (*I*), is dependent on the excitation laser power (*P*) as follows^32^:

2 $$I = AP^{n}$$

where *A* is a proportional parameter, *n* is the number of pumping photons involved in the UC process.

**Figure 6:**
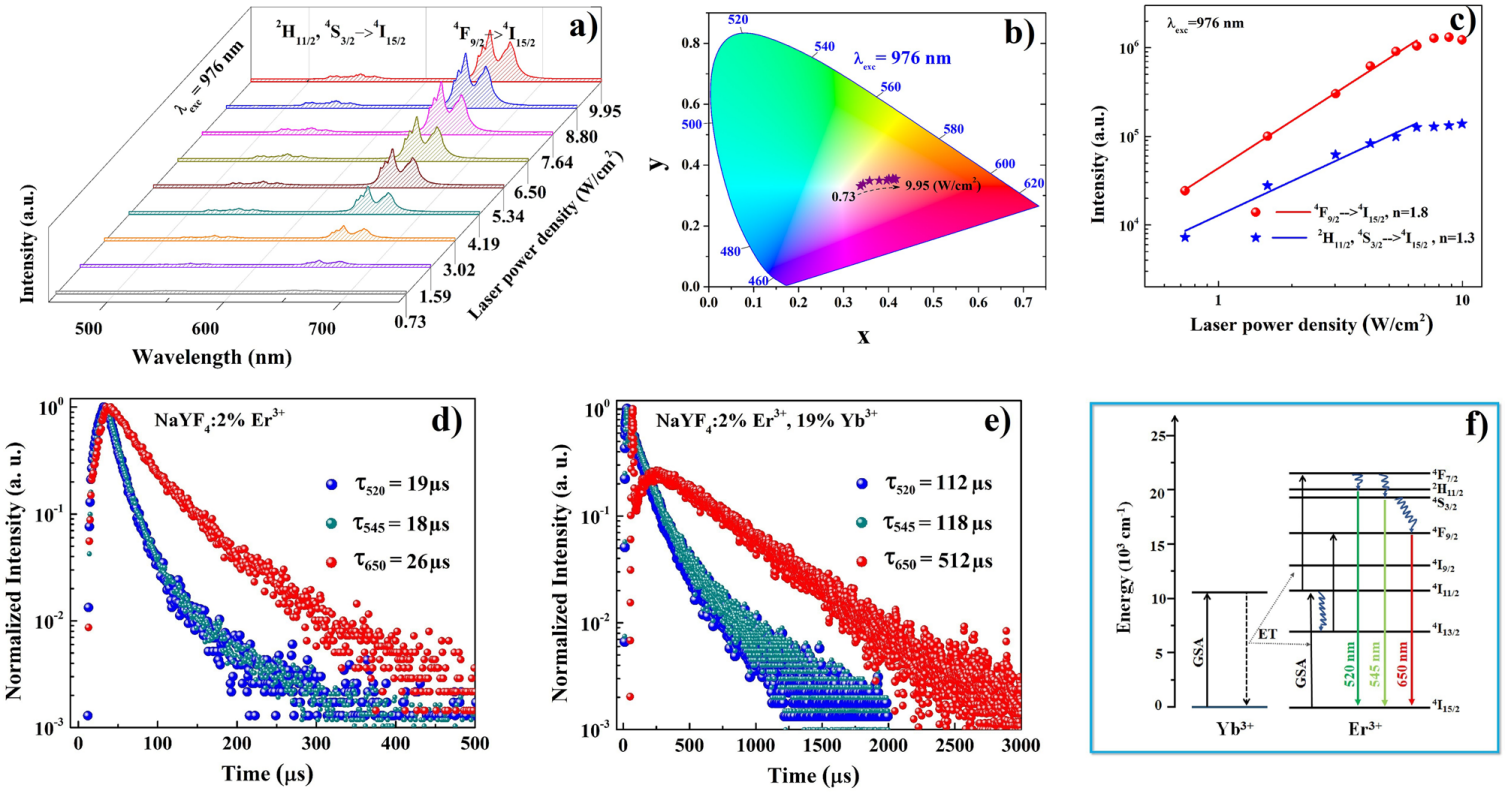
The UC emission spectra (**a**), CIE1931 chromaticity diagram (**b**), and integrated intensities (**c**) of blue (^2^H_11/2_, ^4^S_3/2_ → ^4^I_15/2_) and red (^4^F_9/2_ → ^4^I_15/2_) emission bands of core β-NaYF_4_:2%Er^3+^,19%Yb^3+^ nanoparticles upon 976 nm laser diode excitation in range of 0.73 to 9.95 W/cm^2^. The luminescence decay curves of synthesized β-NaYF_4_:2%Er^3+^ CNP (**d**) and β-NaYF_4_:2%Er^3+^,19%Yb^3+^ CNP (**e**) recorded at 520 nm (blue curves), 545 nm (dark cyan curves) and 650 nm (red curves) under 976 nm excitation. The corresponding energy levels diagram of NaYF_4_:Er^3+^,Yb^3+^ nanoparticles (**f**).

Figure 6 shows the UC emission properties of β-NaYF_4_:2%Er^3+^,19%Yb^3+^ CNP as a function of pump power of 976 nm laser diode. As can be observed, the β-NaYF_4_:2%Er^3+^,19%Yb^3+^ CNP revealed strong red emission band at 650 nm corresponding to ^4^F_9/2_ → ^4^I_15/2_ transition of Er^3+^ ions and minor green emission bands at 520 nm and 545 nm corresponding to ^2^H_11/2_, ^4^S_3/2_ → ^4^I_15/2_ transitions, respectively (Fig. 6a). The UC emission intensity increased with increasing the pump power and the intensity ratio of green to red emission decreased from 0.3 to 0.1 in response to increased excitation power density from 0.73 to 9.95 W/cm^2^. These results suggest that at high excitation densities the red emission is even more dominant. High excitation density provides heating of the nanocrystals, therefore observed changes of green to red emission intensities could be explained in terms of higher probability of nonradiative depopulation of ^2^H_11/2_ and ^4^S_3/2_ states to ^4^F_9/2_ at higher temperatures. The calculated CIE chromaticity coordinates of β-NaYF_4_:2%Er^3+^,19%Yb^3+^ CNP under 976 nm excitation confirmed that the CIE (x, y) values increased from (0.337, 0.331) to (0.409, 0.356) with the excitation power (Fig. 6b). The obtained CIE (x, y) values proved that the emission color of the β-NaYF_4_:2%Er^3+^,19%Yb^3+^ CNP can be tuned from yellowish to red by excitation power changes (Table 1). The calculated slopes of the fitting lines of the first 6 points for green (^2^H_11/2_, ^4^S_3/2_ → ^4^I_15/2_) and red (^4^F_9/2_ → ^4^I_15/2_) emission bands were equal to 1.8 and 1.3, indicating that two photons process was involved to populate excited levels resulting in both green and red emission bands in the synthesized β-NaYF_4_:2%Er^3+^,19%Yb^3+^ CNP (Fig. 6c).

**Table 1:** The integrated UC intensities of particular emission bands, their ratios and CIE chromaticity coordinates of synthesized β-NaYF_4_:Er^3+^,Yb^3+^ CNP.

Laser power density (W/cm^2^)	*Relative integrated intensities*			*CIE*	
	^2^H_11/2_, ^4^S_3/2_ → ^4^I_15/2_	^4^F_9/2_ → ^4^I_15/2_	I_Green_/I_Red_	*x*	*y*
0.73	22.95	77.05	0.30	0.337	0.331
1.59	21.70	78.30	0.28	0.343	0.339
3.02	17.02	82.98	0.21	0.358	0.348
4.19	11.80	88.20	0.13	0.379	0.349
5.34	9.85	90.15	0.11	0.396	0.349
6.50	10.27	89.73	0.11	0.401	0.355
7.64	9.45	90.55	0.10	0.412	0.352
8.80	9.31	90.69	0.10	0.416	0.355
9.95	9.35	90.65	0.10	0.409	0.356

In addition, luminescence decay curves of ^2^H_11/2_ (emission band at 520 nm), ^4^S_3/2_ (emission band at 545 nm) and ^4^F_9/2_ (emission band at 650 nm) excited levels of β-NaYF_4_:2%Er^3+^,19%Yb^3+^ CNP and β-NaYF_4_@NaYF_4_:2%Er^3+^,19%Yb^3+^ CSNP compared with the β-NaYF_4_:2%Er^3+^ CNP and β-NaYF_4_@NaYF_4_:2%Er^3+^ CSNP under 976 nm excitation were recorded. The average luminescence lifetimes were calculated by the following equation^33^:

3 $$\tau = \frac{{\mathop \int \nolimits_{0}^{\infty } tI\left( t \right)dt}}{{\mathop \int \nolimits_{0}^{\infty } I\left( t \right)dt}}$$

where *I(t)* represents the luminescence intensity at time *t*.

The average decay times of β-NaYF_4_:2%Er^3+^,19%Yb^3+^ CNP recorded for ^2^H_11/2_ level (emission band at 520 nm), ^4^S_3/2_ level (emission at 545 nm) and ^4^F_9/2_ level (emission at 650 nm) were equal 112, 118 and 512 µs, respectively (Fig. 6e), while for the β-NaYF_4_:2%Er^3+^ CNP counterpart they were 19, 18 and 26 µs, respectively (Fig. 6d). The longer lifetimes of Er^3+^ ions in the synthesized β-NaYF_4_:2%Er^3+^,19%Yb^3+^ CNP compared with the NaYF_4_:2%Er^3+^ CNP confirmed the occurrence of effective energy transfer from Yb^3+^ to Er^3+^ ions in β-NaYF_4_ host lattice^34^. What is more, the comparison of the intensities of the bands within the β-NaYF_4_:2%Er^3+^,19%Yb^3+^ CNP themselves revealed that green emission intensity was much weaker than the red one and associated lifetimes recorded at 520 and 545 nm was about 4.34 times shorter than that recorded at 650 nm.

Basing on the obtained results, the UC mechanism of the β-NaYF_4_:2%Er^3+^,19%Yb^3+^ CNP have been proposed as follows: upon a 976 nm laser diode excitation, the activator ions (Er^3+^) were excited from the ^4^I_15/2_ ground state to the ^4^I_11/2_ excited state through ground states absorption (GSA), or energy transfer (ET) from the ^2^F_5/2_ excited state of Yb^3+^ ions (4). Then, the population of ^4^I_11/2_ excited state of Er^3+^ ions was followed by excitation to the ^4^F_7/2_ level due to another energy transfer from Yb^3+^ ions (5).

4 $$^{4} {\text{I}}_{15/2} \left( {{\text{Er}}^{3 + } } \right) + {^{2} {\text{F}}_{5/2}} \left( {{\text{Yb}}^{3 + } } \right) \to{^{4} {\text{I}}_{11/2}} \left( {{\text{Er}}^{3 + } } \right) +{^{2} {\text{F}}_{7/2}} \left( {{\text{Yb}}^{3 + } } \right)$$

5 $$^{4} {\text{I}}_{11/2} \left( {{\text{Er}}^{3 + } } \right) +{^{2} {\text{F}}_{5/2}} \left( {{\text{Yb}}^{3 + } } \right) \to{^{4} {\text{F}}_{7/2}} \left( {{\text{Er}}^{3 + } } \right) +{^{2} {\text{F}}_{7/2}} \left( {{\text{Yb}}^{3 + } } \right)$$

After the excitation of ^4^F_7/2_ level of Er^3+^ ions the nonradiative relaxation to the ^2^H_11/2_ and ^4^S_3/2_ levels occurred. The radiative depopulation of those levels back to the ground state leads to appearance of green UC emission bands at 520 and 545 nm corresponding to ^2^H_11/2_ → ^4^I_15/2_ and ^4^S_3/2_ → ^4^I_15/2_ transitions of Er^3+^ ions, respectively (Fig. 6f)^15^. Additionally, the electrons from the ^4^S_3/2_ level of Er^3+^ ions can be nonradiatively relaxed to the ^4^F_9/2_ level causing the occurrence of the red emission band at 650 nm related to the ^4^F_9/2_ → ^4^I_15/2_ electronic transition of Er^3+^ ions. Furthermore, the nonradiative relaxation from ^4^I_11/2_ excited state to the lower ^4^I_13/2_ state of Er^3+^ ions can take place enabling another act of excitation to occur. Therefore, absorption of a second NIR photon can provoke excitation from ^4^I_11/2_ to the ^4^F_9/2_ state^34^.

**Figure 7:**
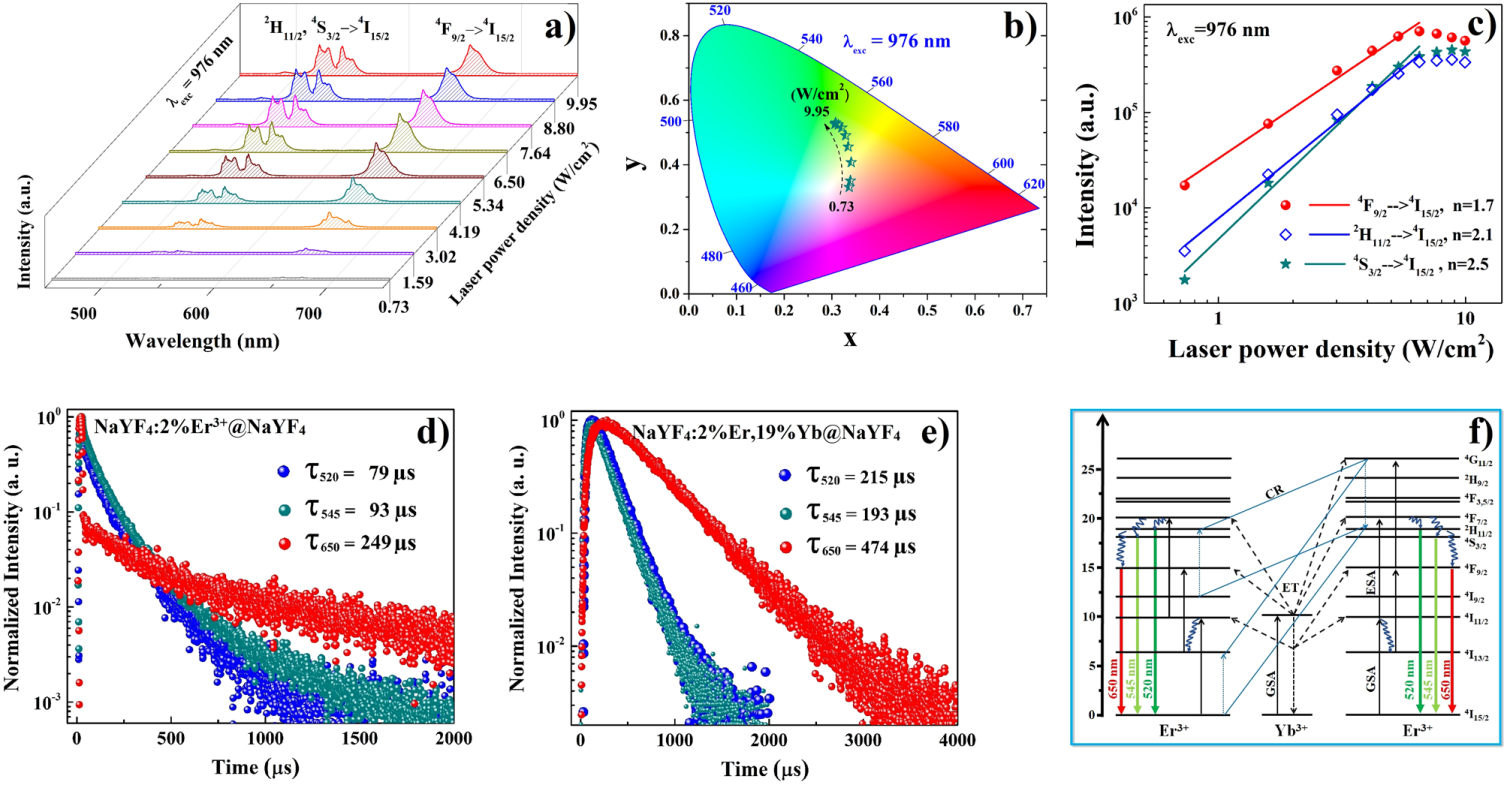
The UC emission spectra (**a**), and CIE chromaticity diagram (**b**), and integrated intensities (**c**) of green (^2^H_11/2_, ^4^S_3/2_ → ^4^I_15/2_, dark green- and blue- lines) and red (^4^F_9/2_ → ^4^I_15/2_, red-line) emission bands of synthesized β-NaYF_4_:2%Er^3+^,19%Yb^3+^@NaYF_4_ CSNP upon 976 nm laser diode excitation in range of 0.73 to 9.95 W/cm^2^. The luminescence decay curves of synthesized β-NaYF_4_:2%Er^3+^@NaYF_4_ (**d**) and β-NaYF_4_:2%Er^3+^,19%Yb^3+^@NaYF_4_ CSNP (**e**) recorded at 520 nm (blue curves), 545 nm (dark cyan curves) and 650 nm (red curves) under 976 nm excitation. The corresponding energy levels diagram of NaYF_4_:Er^3+^,Yb^3+^@ NaYF_4_ nanoparticles (**f**).

In order to study the effect of the shell layer on the UC properties of nanoparticles, an analogous spectroscopic analysis was carried out for β-NaYF_4_:Er^3+^,Yb^3+^@NaYF_4_ CSNP. The UC emission properties of synthesized β-NaYF_4_:Er^3+^,Yb^3+^@NaYF_4_ CSNP upon 976 nm laser diode excitation are presented in Fig. 7. It can be clearly seen that these CSNPs revealed three strong emission bands at 520 nm, 545 nm and 650 nm assigned to ^2^H_11/2_ → ^4^I_15/2_, ^4^S_3/2_ → ^4^I_15/2_ and ^4^F_9/2_ → ^4^I_15/2_ transitions of Er^3+^ ions, respectively (Fig. 7a). The ratio of green to red emission intensities increased from 0.31 to 1.36 with increasing excitation power density from 0.73 to 9.95 W/cm^2^. The calculated CIE chromaticity coordinates of synthesized β-NaYF_4_:2%Er^3+^,19%Yb^3+^@NaYF_4_ CSNP proved that the *x* value decreased from 0.34 to 0.31, while *y* value increased from 0.33 to 0.53 (Table 2) therefore, the emission colors changed from red into yellowish green (Fig. 7b). The slopes of fitting lines for emission bands associated with the transitions from ^4^S_3/2_ (blue line), ^2^H_11/2_ (wine line) and ^4^F_9/2_ (red line) levels to ^4^I_15/2_ ground state were determined as 2.5, 2.1 and 1.7, respectively (Fig. 7c). These results indicate that in the case of synthesized β-NaYF_4_:2%Er^3+^,19%Yb^3+^@NaYF_4_ CSNP not only two-photon UC process, as in the above-mentioned β-NaYF_4_:2%Er^3+^,19%Yb^3+^ CNP took place, but also three-photon UC process for green emissions was involved.

**Table 2:** The integrated UC intensities of particular emission bands, their ratios and CIE chromaticity coordinates of synthesized β-NaYF_4_:Er^3+^,Yb^3+^@NaYF_4_ CSNP.

Laser power density (W/cm^2^)	*Relative integrated intensities*				*CIE*	
	^2^H_11/2_ → ^4^I_15/2_	^4^S_3/2_ → ^4^I_15/2_	^4^F_9/2_ → ^4^I_15/2_	I_Green_/I_Red_	*x*	*y*
0.73	7.83	15.66	76.51	0.31	0.336	0.331
1.59	15.52	19.14	65.34	0.53	0.339	0.351
3.02	18.58	20.97	60.45	0.65	0.341	0.407
4.19	23.33	21.48	55.19	0.81	0.334	0.456
5.34	25.36	21.60	53.04	0.89	0.329	0.490
6.50	26.89	23.61	49.50	1.02	0.321	0.515
7.64	29.67	24.18	46.15	1.17	0.313	0.524
8.80	31.78	25.17	43.06	1.32	0.308	0.530
9.95	32.53	25.12	42.35	1.36	0.306	0.526

The three-photon UC process causing the green emission was observed only in the β-NaYF_4_:2%Er^3+^,19%Yb^3+^@NaYF_4_ CSNP. Therefore it is postulated in this work, that an additional shell layer reduces undesirable surface losses, detain energy within the sample and enable cross-relaxation between Er^3+^ ions to occur. The cross-relaxation processes of two Er^3+^ ions taking place in the way as follows^34,35^:

6 $$^{4} {\text{I}}_{15/2} \left( {{\text{Er}}^{3 + } } \right) +{^{4} {\text{G}}_{11/2}} \left( {{\text{Er}}^{3 + } } \right) \, \to{^{4} {\text{I}}_{13/2}} \left( {{\text{Er}}^{3 + } } \right) \, +{^{2} {\text{H}}_{11/2}} ,{^{4} {\text{S}}_{3/2}} \left( {{\text{Er}}^{3 + } } \right)$$

7 $$^{4} {\text{I}}_{9/2} \left( {{\text{Er}}^{3 + } } \right) +{^{4} {\text{G}}_{11/2}} \left( {{\text{Er}}^{3 + } } \right) \to{^{2} {\text{H}}_{11/2}} \left( {{\text{Er}}^{3 + } } \right) +{^{2} {\text{H}}_{11/2}} ,{^{4} {\text{S}}_{3/2}} \left( {{\text{Er}}^{3 + } } \right)$$

To confirm also the energy transfer between Yb^3+^ to Er^3+^ ions in the core–shell β-NaYF_4_@NaYF_4_ host lattice, the comparison of spectroscopic properties of β-NaYF_4_:2%Er^3+^,19%Yb^3+^@NaYF_4_ CSNP and β-NaYF_4_:2%Er^3+^@NaYF_4_ CSNP was made. As can be seen in Fig. 7d, the calculated emission lifetimes of single doped β-NaYF_4_:2%Er^3+^@NaYF_4_ CSNP monitored at 520 nm, 545 nm and 650 nm were 79, 93 and 249 µs, respectively, while in the case of co-doped β-NaYF_4_:2%Er^3+^,19%Yb^3+^@NaYF_4_ CSNP, an approximately three-fold increase in the decay times of green emissions and two-fold of the red one was observed (Fig. 7e) and their values were 215, 193 and 474 µs, respectively. What is more, it can be seen that the lifetime of red emission recorded at 650 nm in the case of synthesized core–shell β-NaYF_4_:2%Er^3+^,19%Yb^3+^@NaYF_4_ CSNP was slightly shorter than in uncoated β-NaYF_4_:2%Er^3+^,19%Yb^3+^ CNP, however, clear increase in lifetime recorded at green emission (corresponding to ^2^H_11/2_ and ^4^S_3/2_ states) was noted. It matched well with the UC emission spectrum and the calculated slopes for UC green and red emission bands as mentioned above. Such outcomes confirmed the significant influence of NaYF_4_ shell layer on the UC energy transfer mechanism appearing in those samples. The schematic model of the energy transfer mechanisms of β-NaYF_4_:2%Er^3+^,19%Yb^3+^@NaYF_4_ CSNP including cross- relaxation processes is presented in Fig. 7f. The excitation wavelength of 975 nm involved in the experiment is matched to the excitation of the sensitizer Yb^3+^ ions from which energy is transferred to Er^3+^ ions. The energy transfer (ET) from Yb^3+^ to Er^3+^ ions marked with a dashed line in the diagram increases the efficiency of population of ^4^I_11/2_, ^4^F_9/2_ and ^4^I_7/2_ levels, which, as can be seen in the diagram, in our co-doped core–shell structure are involved in both 2-photon and 3-photon upconversion processes. The population of ^4^I_11/2_ level can be followed by two processes, i.e. 1) ^4^I_11/2_ → ^4^F_7/2_ ESA process, followed by rapid relaxation to ^2^H_11/2_, ^4^S_3/2_ and ^4^F_9/2_ levels, from which the green and red emissions, respectively occur and 2) nonradiative population of the lower ^4^I_13/2_ level, from which the next upconversion excitation act to the ^4^F_9/2_ level takes place. The population of the ^4^F_9/2_ level allows direct radiative transition to the ground ^4^I_15/2_ state to occur, resulting in the appearance of red emission at 650 nm. However two other events from this state can also supervene, i.e. nonradiative relaxation to ^4^I_9/2_ and ^4^I_11/2_ levels or a population of higher lying ^4^G_11/2_ level. Both the filling of the ^4^I_11/2_ level followed by excitation to the ^4^F_7/2_ level, as well as the process of cross-relaxation occurring as a result of the filling of the ^4^I_9/2_ and ^4^G_11/2_ levels, lead to subsequent emission transitions thus increasing the efficiency of the observed green emission. What is more, both these processes, i.e. the occurrence of nonradiative relaxation and excitation to the ^4^G_11/2_ level reduce the population of the ^4^F_9/2_ level, which explains the lowering of red emission efficiency in favor of green one.

In this work, we have shown that the coating of the CNP by the additional shell layer reduces the efficiency of the surface-related nonradiative quenching processes resulting in the enhancement of the green emission intensity for CSNP in respect to the CNP. Therefore, the core-sell structure in general improved the intensity of bands and thus also extended the decay times, excluding the red emission in the case of β-NaYF_4_:2%Er^3+^,19%Yb^3+^@NaYF_4_ CSNP, however, such a structure weakened the efficiency of energy transfer.

**Figure 8:**
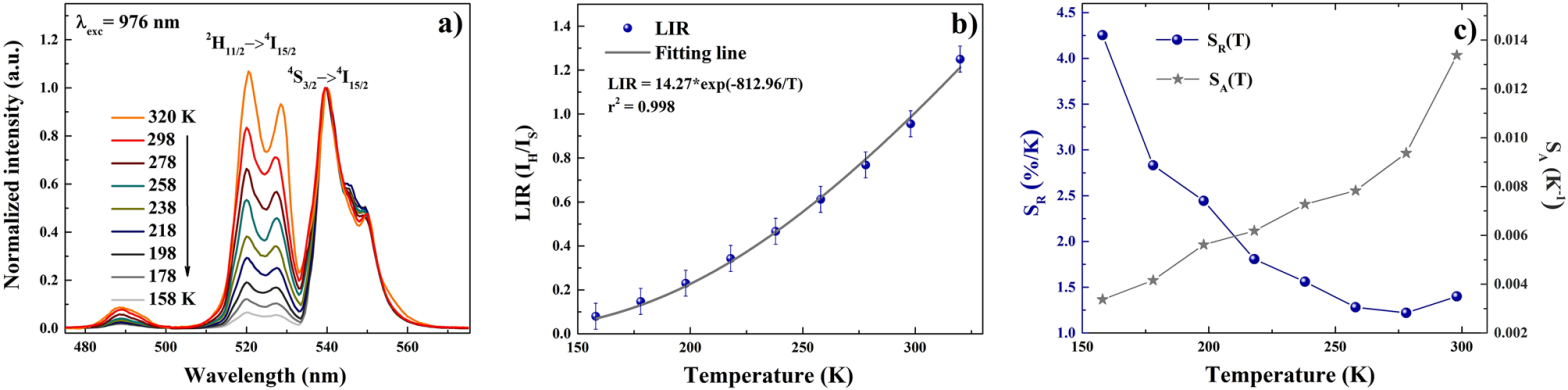
Normalized UC emission spectra of synthesized β-NaYF_4_:2%Er^3+^,19%Yb^3+^@NaYF_4_ CSNP upon 976 nm laser diode excitation measured at temperatures ranging from 158 to 320 K with step of 20 K (**a**), the fluorescence intensity ratio (I_H_/I_S_) of green emission as a function of temperature where the experimental data is marked as points and the line corresponds to the fitting data (**b**), the absolute (gray line and stars) and relative (dark blue line and spherical points) sensitivity as a function of temperature (**c**).

In order to investigate the suitability of β-NaYF_4_:Er^3+^,Yb^3+^@NaYF_4_ CSNP for optical temperature sensing, the temperature dependence of UC emission spectra under the 976 nm excitation (power density 4.29 W/cm^2^) at temperatures ranging from 158 to 320 K was measured (Fig. 8a). As can be clearly seen in emission spectra normalized to the intensity of red band which is presented in the Fig. 8a, the intensity of UC green emission of these CSNP increased with increasing temperature. The luminescence intensity ratio (LIR) related to ^2^H_11/2_ → ^4^I_15/2_ and ^4^S_3/2_ → ^4^I_15/2_ transitions of two thermally coupled excited states can be derived from Boltzmann equation as follow^36^:

8 $$LIR = \frac{{I_{H} }}{{I_{S} }} = \frac{{g_{H}\ A_{H }\ h\nu_{H } }}{{g_{S}\ A_{S }\ h\nu_{S } }}\exp \left( { - \frac{\Delta E}{kT}} \right) = B\exp \left( { - \frac{\Delta E}{kT}} \right)$$

where, I_H_, I_S_, g_H_, g_S_, A_H_, A_S_, ν_H_ and ν_S_ are the integrated emission intensities, degeneracies, spontaneous emission rates and frequencies of the transitions from the ^2^H_11/2_ (or ^4^S_3/2_) state to the ground state, respectively; h is Planck’s constant; k is Boltzmann constant (k ~ 0.695035 cm^−1^/K); T is the temperature, ΔE is energy gap between the ^2^H_11/2_ and ^4^S_3/2_ levels and B is the associated constant of host lattice.

The energy difference between ^2^H_11/2_ and ^4^S_3/2_ levels and the associated constant of β-NaYF_4_ host lattice were determined by fitting the data by exponential function as presented in Fig. 8b. The obtained ΔE/k and B values for fit were equal to ~ 813 cm^−1^ and 14.27 (r^2^ = 0.998), respectively.

To evaluate the sensor performance to temperature sensing, the relative thermal sensitivity (S_R_) of luminescent thermometer has to be determined. Basing on the experimental data of LIR, both the absolute thermal sensitivity (S_A_) and the relative thermal sensitivity (S_R_) of synthesized β-NaYF_4_:Er^3+^,Yb^3+^@NaYF_4_ CSNP was calculated by the following equations^36^:

9 $$S_{A} = \frac{\Delta (LIR)}{{\Delta T}}$$

10 $$S_{R} = \frac{1}{LIR}\frac{\Delta (LIR)}{{\Delta T}}$$

The results are presented in the Fig. 8c and as can be seen, the relative thermal sensitivity (dark blue line and spherical points) of β-NaYF_4_:Er^3+^,Yb^3+^@NaYF_4_ CSNP was decreasing from unprecedented value S_R_ = 4.25% K^−1^ at 158 K with increasing temperature, however maintaining high value > 1% K^−1^ in the whole analyzed temperature range. On the other hand, the absolute thermal sensitivity (gray line and stars) revealed opposite thermal behavior and increased in this temperature range reaching values of 0.337% K^−1^ at 158 K and 1.338% K^−1^ at 298 K.

## Conclusions

In conclusion, the β-NaYF_4_:Er^3+^,Yb^3+^@NaYF_4_ CSNP were successfully synthesized by soft template method. The size, shape, crystallinity and UC emission properties of this material were investigated and compared with uncoated β-NaYF_4_:Er^3+^,Yb^3+^ CNP. The results obtained shown that the synthesized β-NaYF_4_:Er^3+^,Yb^3+^@NaYF_4_ CSNP were about 100 ± 10 nm in size, therefore, they were nearly 2.5 times bigger than the β-NaYF_4_:Er^3+^,Yb^3+^ CNP. The UC emission spectra measured at room temperature under 976 nm excitation of power density in range from 0.73 to 9.95 W/cm^2^ demonstrated that the synthesized NaYF_4_:Er^3+^,Yb^3+^@NaYF_4_ CSNP compared with β-NaYF_4_:Er^3+^,Yb^3+^ CNP revealed both the stronger UC emission intensity and the 13 times greater the intensity ratio of green to red emission. The calculated CIE (x, y) values have proven the predominance of red color emission in the case of β-NaYF_4_:2%Er^3+^,19%Yb^3+^ CNP and the shift from red color into yellowish green area in the case of NaYF_4_:2%Er^3+^,19%Yb^3+^ @NaYF_4_ CSNP. The calculated values of slopes of green and red emission intensities versus excitation power density also confirmed that in the NaYF_4_:2%Er^3+^,19%Yb^3+^@NaYF_4_ CSNP not only two-photon UC process as in the case of β-NaYF_4_:2%Er^3+^,19%Yb^3+^ CNP occurred, but also three-photon UC process, due to the cross-relaxation existing between Er^3+^ ions took place, enabling green emission intensity enhancement. The calculated lifetimes of ^2^H_11/2,_^4^S_3/2_ and ^4^F_9/2_ excited levels of Er^3+^ ions within single doped β-NaYF_4_:2%Er^3+^ CNP and β-NaYF_4_@NaYF_4_:2%Er^3+^ CSNP were shorter than that of co-doped β-NaYF_4_:2%Er^3+^,19%Yb^3+^ CNP and β-NaYF_4_@NaYF_4_:2%Er^3+^,19%Yb^3+^ CSNP, which confirmed that energy transfer from Yb^3+^ to Er^3+^ ions in synthesized materials occurred. What is more, the comparison of β-NaYF_4_:2%Er^3+^,19%Yb^3+^ CNP and β-NaYF_4_:2%Er^3+^,19%Yb^3+^@NaYF_4_ CSNP showed that the lifetime of ^4^F_9/2_ excited level associated with red emission band recorded at 650 nm in the case of synthesized core–shell structures was slightly shorter than in uncoated nanoparticles, however, clear increase in lifetime recorded at green emission (corresponding to ^2^H_11/2_ and ^4^S_3/2_ states) was noted which confirmed the significant influence of NaYF_4_ shell on the UC energy transfer mechanisms occurring in core–shell nanomaterials.

The temperature dependent UC emission spectra of β-NaYF_4_:2%Er^3+^,19%Yb^3+^@NaYF_4_ CSNPs under the 976 nm laser diode excitation of power density of 4.29 W/cm^2^ have been investigated to evaluate their suitability for temperature sensing applications. Basing on the LIR values of emission bands of two thermally coupled excited states of Er^3+^ ions, the energy gap between ^2^H_11/2_ and ^4^S_3/2_ levels and associated constant of NaYF_4_@NaYF_4_ host lattice were determined and they were equal to ~ 813 cm^−1^ and 14.27 (r^2^ = 0.998), respectively. The relative thermal sensitivity of β-NaYF_4_:2%Er^3+^,19%Yb^3+^@NaYF_4_ CSNPs determined on the basis of the LIR achieved unusually high value of S_R_ = 4.25% K^−1^ at 158 K and was decreasing with increasing temperature, however, maintaining value > 1% K^−1^ in the whole analyzed temperature range, up to 298 K. These outcomes confirmed that NaYF_4_:2%Er^3+^,19%Yb^3+^@NaYF_4_ CSNPs reveal very high potential for temperature sensing applications.

## References

[1] Childs PRN, Greenwood JR, Long CA (2000). Review of temperature measurement. Rev. Sci. Instrum..

[2] Mecklenburg M, Hubbard WA, White ER, Dhall R, Cronin SB, Aloni S, Regan BC (2015). Nanoscale temperature mapping in operating microelectronic devices. Science.

[3] Yu YB, Chow WK (2009). Review on an advanced high-temperature measurement technology: the optical fiber thermometry. J. Thermodyn..

[4] Sun, P. S., Sun, M., Tang, Y. Q., Yang, S., Pang, T., Zhang, Z. R. & Dong, F. Z., Measurement of temperature distribution based on optical fiber-sensing technology and tunable diode laser absorption spectroscopy. *Temperature Sensing chapter 3* (Edited by Ivanka Stanimirović, Zdravko Stanimirović, Intech Open, Lndon, United Kingdom) 10.5772/intechopen.76687 (2018).

[5] Brites CDS, Lima PP, Silva NJO, Millán A, Amaral VS, Palacio F, Carlos LD (2012). Thermometry at the nanoscale. Nanoscale.

[6] Wang, X., Wang, Y., Marques-Hueso, J. & Yan, X. (2017) Improving optical temperature sensing performance of Er3+ doped Y2O3 microtubes via co-doping and controlling excitation power. *Sci. Rep.* **7**, 758(1–13). 10.1038/s41598-017-00838-w . 10.1038/s41598-017-00838-w PMC5429693 28389639

[7] Abram C, Fond B, Beyrau F (2018). Temperature measurement techniques for gas and liquid flows using thermo graphic phosphor tracer particles. Prog. Energy Combust. Sci..

[8] Marciniak L, Bednarkiewicz A (2016). The influence of dopant concentration on temperature dependent emission spectra in LiLa1-x-yEuxTbyP4O12 nanocrystals: toward rational design of highly-sensitive luminescent nanothermometers. Phys. Chem. Chem. Phys..

[9] Lu H, Hao H, Gao Y, Li D, Shi G, Song Y, Wang Y, Zhang X (2017). Optical sensing of temperature based on non-thermally coupled levels and upconverted white light emission of a Gd2(WO4)3 phosphor co-doped with in Ho(III), Tm(III), and Yb(III). Microchim. Acta.

[10] Geitenbeeka RG, Salzmanna BBV, Nieuwelink AE, Meijerink A, Weckhuyse BM (2019). Chemically and thermally stable lanthanide-doped Y2O3 nanoparticles for remote temperature sensing in catalytic environments. Chem. Eng. Sci..

[11] Sharma RK, Mudring AV, Ghosh P (2017). Recent trends in binary and ternary rare-earth fluoride nanophosphors: How structural and physical properties influence optical behavior. J. Lumin..

[12] Tan M, Rosal B, Zhang Y, Rodríguez EM, Hu J, Zhou Z, Fan R, Ortgies DH, Fernández N, Chaves-Coira I, Núñez A, Jaque D, Chen G (2018). Rare-earth-doped fluoride nanoparticles with engineered long luminescence lifetime for time-gated in vivo optical imaging in the second biological window. Nanoscale.

[13] Wang M, Abbineni G, Clevenger A, Mao C, Xu S (2011). Upconversion nanoparticles: synthesis, surface modification, and biological applications. Nanomedicine.

[14] Li H, Lei W, Wu J, Li S, Zhou G, Liu D, Yang X, Wang S, Li Z, Zhang J (2018). An upconverting nanotheranostic agent activated by hypoxia combined with NIR irradiation for selective hypoxia imaging and tumour therapy. J. Mater. Chem. B.

[15] Peng, Y. P., Lu, W., Ren, P., Ni, Y., Wang, Y., Yan, P., Zeng, Y. J., Zhang, W. & Ruan, S., Multi-band up-converted lasing behavior in NaYF_4_:Yb/Er Nanocrystals. *Nanomaterials* **8**, 497 (1–10). 10.3390/nano8070497 (2018). 10.3390/nano8070497 PMC6071127 29976916

[16] Liu S, Tian B, Wu S, Wang Y, Huang J, Gao B, Jin L, Li K, Wang Z (2018). pH-sensitive polymergated multifunctional upconversion NaYF_4_:Yb/Er@mSiO_2_ nanocomposite for oral drug delivery. Microporous Mesoporous Mater..

[17] Li, Z., Miao, H., Fu, Y., Liu, Y., Zhang, R. and Tang, B., Fabrication of NaYF_4_:Yb,Er nanoprobes for cell imaging directly by using the method of hydrion rivalry aided by ultrasonic. *Nanoscale Res. Lett.* **11**, 441(1–10). 10.1186/s11671-016-1651-y (2016) 10.1186/s11671-016-1651-y PMC5045454 27696322

[18] Tang J, Lei L, Feng H, Zhang H, Han Y (2016). Preparation of K^+^-doped core-shell NaYF_4_:Yb, Er upconversion nanoparticles and its application for fluorescence immunochromatographic assay of human procalcitonin. J. Fluoresc..

[19] Shota, S., Umezawa, M., Kuraoka, S., Ube, T., Kamimura, M. & Soga, K., Temperature sensing of deep abdominal region in mice by using over-1000 nm near-infrared luminescence of rare-earth-doped NaYF_4_ nanothermometer. *Sci. Rep. 8*, 6979(1–12). 10.1038/s41598-018-35354-y (2018) 10.1038/s41598-018-35354-y PMC6242879 30451921

[20] Dai Y, Yang D, Ma P, Kang X, Zhang X, Li C, Hou Z, Cheng Z, Lin J (2012). Doxorubicin conjugated NaYF_4_:Yb^3+^/Tm^3+^ nanoparticles for therapy and sensing of drug delivery by luminescence resonance energy transfer. Biomaterials.

[21] Leipeng L, Feng Q, Yuan Z, Yangdong Z, Hua Z, Zhiguo Z (2019). Study on the thermal sensitivity of β-NaYF_4_:Yb^3+^-Er^3+^ nano-thermometers based on luminescence ratiometric technology. Curr. Appl. Phys..

[22] Wortmann L, Suyari S, Ube T, Kamimura M, Soga K (2018). Tuning the thermal sensitivity of β-NaYF_4_: Yb^3+^, Ho^3+^, Er^3+^ nanothermometers for optimal temperature sensing in OTN-NIR (NIR II/III) biological window. J. Lumin..

[23] Tong L, Li X, Zhang J, Xu S, Sun J, Zheng H, Zhang Y, Zhang X, Hua R, Xia H, Chen B (2017). NaYF_4_:Sm^3+^/Yb^3+^@NaYF_4_:Er^3+^/Yb^3+^ core-shell structured nanocalorifier with optical temperature probe. Opt. Express.

[24] Klier DT, Kumke MU (2015). Upconversion luminescence properties of NaYF_4_:Yb: Er nanoparticles codoped with G^d3^+. J. Phys. Chem. C.

[25] Geitenbeek RG, Prins PT, Albrecht W, Blaaderen A, Weckhuysen BM, Meijerink A (2017). NaYF_4_:Er^3+^, Y^b3^+/Si_O_2 Core/shell upconverting nanocrystals for luminescence thermometry up to 900 K. J. Phys. Chem. C.

[26] Li X, Yang L, Zhu Y, Zhong J, Chen D (2019). Upconversion of transparent glass ceramics containing β-NaYF_4_:Yb^3+^, E^r3^+ nanocrystals for optical thermometry. RSC. Adv..

[27] Cui Y, Meng Q, Lü S, Sun W (2019). Temperature sensing properties base on up-conversion luminescence for NaYF_4_:Er^3+^, Yb^3+^ phosphor. Chem. Sel..

[28] Giang LTK, Anh TK, Binh NT, Minh LQ (2015). Fabrication and upconversion emission processes in nanoluminophores NaYF4:Er, Yb and NaYF4:Tm, Yb. Int. J. Nanotechnol..

[29] Chatterjee, A. K. Chapter 8: X-ray diffraction. In *Handbook of Analytical Techniques in Concrete Science and Technology: Principles, Techniques, and Applications* (Elsevier, 2001). Hardcover ISBN: 9780815514374. 10.1016/B978-081551437-4.50011-4.

[30] Yadav, L. D. S. Chapter 3: Infrared (IR) Spectroscopy. In *Organic Spectroscopy* 52–106 (Springer, Dordrecht, 2005). Print ISBN 978-94-017-2508-8. 10.1007/978-1-4020-2575-4.

[31] Boschetto F, Toyama N, Horiguchi S, Bock RM, Mcentire BJ, Adachi T, Marin E, Zhu W, Mazda O, Bal BS, Pezzotti G (2018). In vitro antibacterial activity of oxide and non-oxide bioceramics for arthroplastic devices: II. Fourier transform infrared spectroscopy. Analyst.

[32] Suyver, J. F., Aebischer, A., García-Revilla, S., Gerner, P. & Güdel, H. U., Anomalous power dependence of sensitized upconversion luminescence. *Phys. Rev. B* **71**(12), 125123(1–9). 10.1103/PhysRevB.71.125123 (2005).

[33] Xu S, Wang Z, Li P, Li T, Bai Q, Sun J, Yang Z (2017). Single-phase white-emitting phosphors Ba_3_Ce_(__1–x−y)_(PO_4_)_3_:xTb^3+^, yM^n2^+ and B_a_3C_e(1–x−z_)(P_O_4_)_3:xT^b3^+, z^Sm^3+: structure, luminescence, energy transfer and thermal stability. RSC Adv..

[34] Dong H, Sun LD, Yan CH (2015). Energy transfer in lanthanide upconversion studies for extended optical applications. Chem. Soc. Rev..

[35] Li, F., Li, J., Chen, L., Huang, Y., Peng, Y., Luo, Y., Zhang, L. & Mu, J., Hydrothermal synthesis and upconversion properties of about 19 nm Sc_2_O_3_:Er^3+^,Yb^3+^ nanoparticles with detailed investigation of the energy transfer mechanism. *Nanoscale Res. Lett.* **13**, 372(1–9). 10.1186/s11671-018-2794-9 (2018). 10.1186/s11671-018-2794-9 PMC6250604 30467782

[36] Siaï A, Haro-González P, Horchani Naifer K, Férid M (2018). Optical temperature sensing of Er^3+^/Yb^3+^ doped LaGdO_3_ based on fluorescence intensity ratio and lifetime thermometry. Opt. Mater..

